# The association between history of retained placenta and success rate of misoprostol treatment for early pregnancy failure

**DOI:** 10.1186/s12905-023-02666-9

**Published:** 2023-10-04

**Authors:** Adiel Cohen, Einat Gutman-Ido, Gilad Karavani, Alon Albeck, Joshua I. Rosenbloom, Asher Shushan, Henry H. Chill

**Affiliations:** 1 Department of Obstetrics and Gynecology, Hadassah Medical Organization, Faculty of Medicine, Hebrew University of Jerusalem, Ein Kerem, Jerusalem, P.O.B. 12000, 91120 Israel; 2 Department of Internal Medicine, Hadassah Medical Organization, Faculty of Medicine, Hebrew University of Jerusalem, Jerusalem, Israel; 3 Division of Urogynecology, University of Chicago Pritzker School of Medicine, NorthShore University HealthSystem, Skokie, IL USA

**Keywords:** Early pregnancy failure, First trimester miscarriage, Missed abortion, Blighted ovum, Retained placenta, retained products of conception

## Abstract

**Background:**

To date, the association between retained placenta and treatment success rate of misoprostol for early pregnancy failure has yet to be evaluated. The aim of this study was to evaluate this association and further investigated the connection between medical, clinical and sonographic parameters and treatment success.

**Methods:**

We conducted a retrospective cohort study of women with early pregnancy failure treated with misoprostol from 2006 to 2021. The success rate of misoprostol treatment was compared between patients with history of retained placenta including women who underwent manual lysis of the placenta following delivery or patients who were found to have retained products of conception during their post-partum period (study group) and patients without such history (controls). Demographic, clinical, and sonographic characteristics as well as treatment outcomes were compared between the groups.

**Results:**

A total of 271 women were included in the study (34 women in the study group compared to 237 women in the control group). Two-hundred and thirty-three women (86.0%) presented with missed abortion, and 38 (14.0%) with blighted ovum. Success rates of misoprostol treatment were 61.8% and 78.5% for the study and control groups, respectively (p = 0.032). Univariate analysis performed comparing successful vs. failed misoprostol treatment showed advanced age, gravidity, parity and gestational sac size (mm) on TVUS were associated with higher misoprostol treatment failure rate. Following a multivariate logistic regression model these variables did not reach statistical significance.

**Conclusion:**

Women who have an event of retained placenta following childbirth appear to have decreased success rate of treatment with misoprostol for early pregnancy failure. Larger studies are needed to confirm this finding.

## Background

Early pregnancy failure (EPF) is estimated to occur in 10–20% of all clinical pregnancies and is one of the most common complications of early pregnancy [1]. While traditionally surgical intervention, i.e. dilatation and curettage (D&C) was considered to be the preferred treatment option, over the past two decades, medical treatment has become increasingly popular showcasing high efficacy and safety with few adverse events [2–6]. Moreover, medical treatment does not appear to impair future fertility potential [7, 8]. Several studies have compared medical treatment to surgical intervention for treatment of EPF showing favorable results [9–11]. This has led to a shift in paradigm and nowadays medical treatment is considered the primary approach for treatment of EPF.

The common medical treatment for EPF is misoprostol (PGE1) which can be administered orally, buccally or vaginally. Side effects are for the most part mild with few reports of major adverse events [10]. Advantages of vaginal administration compared to the oral route include decreased side effects such as nausea, diarrhea, vomiting and abdominal pain [12].

Success rate of treatment with misoprostol for EPF has been reported to range from 50 to 84% [13–15]. Previous studies have attempted to discover variables which may affect treatment efficacy. Dosage, route of administration, gestational age, gestational sac size and fetal size have all been suggested as factors which may influence treatment success [16–19].

Retained placenta affects 0.5-3% of all vaginal deliveries [20]. This clinical scenario is associated with substantial morbidity for women following vaginal delivery mostly due to post-partum hemorrhage. Several mechanisms have been proposed to explain retained placenta such as failed myometrial contraction and partial placenta accreta preventing placental detachment [20, 21]. Notably, treatment with misoprostol has not previously been shown to increase risk of placental complications [22]. Instead, it is plausible that there may be similar mechanisms, such as abnormal implantation or impaired uterine contractility, that may be associated with both failure of medical treatment of EPF and retained placenta after vaginal delivery. However, to date, the association between retained placenta and treatment success rate of misoprostol for EPF has yet to be evaluated.

The aim of this study was to test the hypothesis that patients with a history of complete or partial retained placenta were more likely to have failed medical management of EPF compared to women without a history of retained placenta. We further investigated the connection between medical, clinical and sonographic parameters and treatment success.

## Methods

We conducted a retrospective cohort study at a tertiary university medical center from 2006 to 2021. Institutional ethical review board approval was received (0627-22-HMO).

The cohort included women up to 13 weeks gestation diagnosed with EPF who underwent medical management. Women with a case of manual placental extraction or uterine cavity inspection at time of delivery or retained placenta with need for medical intervention post-partum in a previous pregnancy comprised the study group. The control group included cases collected for a previous study we conducted, comprised of women who underwent treatment with misoprostol for EPF without history of retained placenta between January 2011 and June 2012. Excluded from both groups were non-singleton pregnancies, cases of scar pregnancy, patients with allergy to prostaglandins and women who did not complete the full protocol of medical treatment.

Early pregnancy failure was defined as missed abortion or blighted ovum up to 13 weeks gestation. Diagnosis of missed abortion was made according to the criteria established by Doubilet et al. [23]. Crown rump length (CRL) of 7 mm and mean sac diameter of 25 mm with the absence of cardiac activity on trans-vaginal ultrasound (TVUS) were considered indicative of missed abortion. Further criteria included absence of a visible heartbeat over 2 weeks after a scan showing a gestational sac without a yolk sac or over 11 days after a scan showing a gestational sac with a yolk sac [23].

Confirmation of EPF was established by TVUS scan performed in our medical center reviewed by a specialist in obstetrics and gynecology. According to our routine clinical pathway, following diagnosis each woman was offered one of three options: expectant management, surgical treatment (dilatation and curettage) or medical treatment with vaginal misoprostol. Women were counselled regarding benefits and risks of each option. Those who opted for medical treatment with vaginal misoprostol were eligible for inclusion in the study.

Our department’s protocol for administration of misoprostol has been described previously [24]. In short, following examination by a gynecologist and receipt of informed consent, 800 mcg of misoprostol (4 tablets of Cytotec 200 mcg each) are placed in the posterior vaginal fornix. All patients are asked to present again to the hospital 48–72 h after treatment for an ultrasound scan. If this ultrasound shows a gestational sac or endometrial thickness of above 20 mm, an additional dose of 800 mcg of misoprostol is administered in the same fashion described above. Patients are then instructed to undergo another ultrasound after two weeks or after the next period and if there is still evidence of retained products of conception, they are referred for surgical management. According to our department’s protocol, when the gestational sac is present, women are referred to undergo D&C as opposed to cases of increased endometrial thickness for which operative hysteroscopy is the treatment of choice.

Women in the study group had at least one case of retained placenta following delivery during a previous pregnancy. Retention of placental products included the need for manual placental removal, partial placental evacuation requiring manual inspection of the uterine cavity or retained products of conception diagnosed during the post-partum period. Patients with any one of these clinical scenarios were eligible for inclusion in our study group. Diagnosis of placental retention was made by the most senior physician in the labor and delivery ward at the time. Diagnosis of post-partum retained products of conception was confirmed with pelvic ultrasound.

Data were retrieved from the electronic medical record and included demographic, clinical and sonographic characteristics such as age, gravidity, parity, previous miscarriage, previous treatment with misoprostol, size of gestational sac, fetal crown-rump length (CRL), presence of fetal heart rate on ultrasound prior to diagnosis of EPF, uterine position and treatment success.

The primary outcome was successful medical treatment compared between women who had an event of retained placenta and women who did not. Successful treatment was defined as endometrial thickness of less than 20 mm with no evidence of retained products of conception after one or two doses of misoprostol.

### Statistical analysis

To compare the study covariates chi-square and Fischer exact test were used for categorical variables and the Mann-Whitney test for continuous variables- all distributions were different from normal. Logistic regression was used for multivariate analysis adjusting for available baseline characteristics. The statistical software package SPSS 24.0 (SPSS Inc., Chicago, IL) was used for all data analyses. We report odds ratios (OR), 95% confidence interval (CI), and two-sided P values. P-values < 0.05 were considered statistically significant.

## Results

A total of 271 patients with EPF were included in the study. Mean age and parity of participants were 32.7 ± 6.5 and 1.9 ± 2.2, respectively. Two-hundred and thirty-three women (86.0%) presented with missed abortion, and 38 (14.0%) with blighted ovum. Success rate after treatment with misoprostol was 76.4%.

The study group included 34 women diagnosed with EPF who had a previous case of retained placenta. The control group included 237 women diagnosed with EPF treated with misoprostol without such an event. A comparison of demographic and obstetric characteristics, as well as miscarriage related parameters are summarized in Tables 1 and 2. Women in the study group had higher parity, gravidity, were less likely to have presence of embryonic heartbeat and had a smaller gestational sac size length (mm) on TVUS. Regarding the primary outcome, success rate after treatment with misoprostol was found to be lower in the study group compared to the control group (61.8% vs. 78.5%, p = 0.032).

**Table 1:** Demographic and obstetric characteristics of the study population – patients with and without history of retained placenta

Parameter	No retained placenta (n = 237)	Retained placenta (n = 34)	P value
Age	32.6 ± 6.5	32.8 ± 5.1	0.90
Gravidity	3.6 ± 2.8	4.7 ± 2.7	0.02
Parity	1.9 ± 2.1	2.8 ± 2.1	0.02
Number of past CS	0.3 ± 0.8	0.3 ± 0.6	0.87
Past missed abortion	0.7 ± 1.2	0.9 ± 1.3	0.32
Past D&C	0.25 ± 0.64	0.38 ± 0.82	0.28
Uterine position			0.93
Anteverted	122/161 (75.8)	10/13 (76.9)	
Retroverted	39/ 161 (24.2)	3/13 (23.1)	

Data presented as mean ± SD, n(%) or n/N (%)

*Note*: CS, cesarean section; D&C, dilatation and curettage

**Table 2:** Current early pregnancy failure characteristics and treatment outcomes

Parameter	No retained placenta (n = 237)	Retained placenta (n = 34)	P value
Method of conception			0.086
Spontaneous	208/235 (88.5)	31/33 (93.9)	
IUI	5/235 (2.1)	2/33 (6.1)	
IVF	22/235(9.4)	0 (0)	
Week of gestation	9.4 ± 2.0	9.4 ± 1.7	0.99
Week of gestation by US	6.8 ± 1.3	6.8 ± 1.2	0.98
Diagnosis			0.90
Missed abortion	204 (86.1)	29 (85.3)	
Blighted ovum	33 (13.9)	5 (14.7)	
Presence of gestational sac	229/232 (98.7)	32/32 (100)	0.52
Presence of yolk sac	102/208 (49.0)	21/32 (65.6)	0.081
Presence of embryo	160/230 (69.6)	24/32 (75.0)	0.53
Presence of embryonic pulse prior to diagnosis of EPF	26/232 (11.2)	0 (0)	0.046
Gestational sac size z axis-mm	20.8 ± 12.1	22 ± 15.2	0.81
Gestational sac size width-mm	23.5 ± 13.6	27.2 ± 15.4	0.36
Gestational sac size length-mm	32.5 ± 18.5	24.9 ± 15.6	0.037
Embryo size according to CRL	8.9 ± 0.7	10.3 ± 1.7	0.47
Uterine size (weeks)	7.2 ± 0.11	7.3 ± 0.40	0.83
Bleeding at diagnosis	74 (31.2)	8/33 (24.2)	0.41
Misoprostol treatment success	186 (78.5)	21 (61.8)	0.032
Of patients with success- number of doses of misoprostol			0.035
One	138/186 (74.2)	11/21 (52.4)	
Two	48/186 (25.8)	10/21 (47.6)	

Data presented as mean ± SD, n(%) or n/N (%)

*Note*: IUI, intrauterine insemination; IVF, in-vitro fertilization; US, ultrasound; CRL, crown-rump length, EPF, early pregnancy failure

In the attempt to identify factors affecting misoprostol treatment failure, a univariate analysis was performed comparing successful vs. failed misoprostol treatment (Table 3). Advanced age, gravidity, parity, and gestational sac size length (mm) were associated with higher misoprostol treatment failure rate.

**Table 3:** Demographic and clinical characteristics of the study population – patients with success vs. failure of misoprostol treatment

Parameter	Success (n = 207)	Failure (n = 64)	P
Age	32.2 ± 6.2	34.11 ± 6.6	0.036
Gravidity	3.5 ± 2.5	4.4 ± 3.3	0.026
Parity	1.8 ± 1.9	2.6 ± 2.9	0.009
Number of past CS	0.32 ± 0.82	0.31 ± 0.66	0.98
Past miscarriages	0.71 ± 1.2	0.78 ± 1.2	0.67
Past misoprostol courses	0.09 ± 0.3	0.12 ± 0.57	0.61
Past D&C	0.25 ± 0.65	0.32 ± 0.74	0.53
Uterine position			0.28
Anteverted	103/139 (74.1)	29/35 (82.9)	
Retroverted	36/139 (25.9)	6/35 (17.1)	
Week of gestation	9.4 ± 1.8	9.4 ± 2.5	0.92
Week of gestation by US	6.8 ± 1.5	6.8 ± 1.2	0.81
Diagnosis			0.10
Missed abortion	174 (84.1)	59 (92.2)	
Blighted ovum	33 (15.9)	5 (7.8)	
Presence of gestational sac	200/203 (98.5)	61/61 (100)	0.34
Presence of yolk sac	89/183 (48.6)	34 /57 (59.7)	0.15
Presence of embryo	139/201 (69.2)	45/61 (73.8)	0.49
Presence of embryonic pulse prior to diagnosis of EPF	21/203 (10.3)	5/61 (8.2)	0.62
Gestational sac size length-mm	23.5 ± 11.7	33 ± 3.2	< 0.001
Embryo size according to CRL	8.9 ± 7.9	9.5 ± 10.1	0.72
Uterine size (weeks)	7.3 ± 1.3	7.2 ± 1.4	0.76
Bleeding at diagnosis	65 (31.4)	17 (26.6)	0.50

Data presented as mean ± SD, n(%) or n/N (%)

Note: CS, cesarean section; D&C, dilation and curettage; US, ultrasound; CRL, Crown-rump length; EPF, early pregnancy failure

A logistic regression multivariate analysis was performed in order to identify factors associated with misoprostol treatment failure (Table 4). Advanced age, parity and history of manual lysis or retained placenta entered the logistic regression model but did not reach statistical significance.

**Table 4:** Multivariate logistic regression analysis for misoprostol treatment failure

Parameter	OR	Lower 95% CI	Upper 95% CI	P value
Age	0.96	0.91	1.01	0.139
Parity	0.89	0.78	1.02	0.098
History of manual lysis/retained placenta	2.09	0.96	4.54	0.061

## Discussion

In this study we found that women with EPF who have a history of retained placenta were at increased risk of misoprostol treatment failure. However, following multivariate analysis this finding as well as other parameters evaluated were no longer statistically significant.

Retained placenta following delivery may be complete or partial. Treatment of complete retained placenta entails manual removal of the placenta while partial retention requires uterine cavity examination to remove remaining placental tissue. When retained products of conception are diagnosed during the post-partum period, operative hysteroscopy is often the treatment of choice.

Several mechanisms have been suggested for retained placenta. One of these includes dysfunctional contractility in which the retro-placental myometrium contracts inadequately leading to failure of placental separation during the third stage of labor [25, 26]. Misoprostol treatment for EPF causes uterine contractions which often are sufficient to achieve complete evacuation of uterine content. If uterine contractility is impaired, this may decrease its ability to evacuate products of conception adherent to the uterine wall. Although during the first trimester the uterus is smaller with diminished contractile potential, inherent contractile dysfunction may play a role in failure of misoprostol during treatment of EPF.

Another possible mechanism for retained placenta is abnormally invasive placenta which incorporates a spectrum of clinical scenarios such as adherent placenta and placenta accreta. In this situation spontaneous placental detachment is inhibited leading to partial or whole placental retention. First trimester abortion complicated by placenta accreta is a rare event described in several case reports [27, 28]. In recent years the concept of partial placenta accreta has gained recognition during which only part of the placenta may be pathologically adherent [29]. This phenomenon could explain cases in which only part of the placenta remains intra-uterine following delivery. Such partial invasive attachment may exist during the first trimester, possibly hindering success of medical treatment for EPF, though this hypothesis requires further investigation.

To our knowledge this is the first study to assess the association between retained placenta and success rate of misoprostol treatment for EPF. Other strengths of the study include systematic collection of data, demographic as well as sonographic parameters, a relatively large control group and substantial clinical relevance for caregivers treating women with EPF.

However, apart from its retrospective nature the study has several limitations. The small study group may have caused certain parameters to fall short of reaching statistical significance. The control group was collected during a relatively short time period compared to the study group. However, we believe this had little effect on the results given that treatment protocols and routine follow-up have not changed in our department during the study period.

## Conclusion

In Conclusion, in this study we found women who had an event of retained placenta to have decreased success rate of treatment with misoprostol for EPF. However, this difference was no longer significant following multivariate analysis. While we appreciate the results found here are preliminary, we believe they may help clinicians counsel women deliberating which treatment course to take following diagnosis of EPF. Future studies focusing on this clinical scenario are needed and will most certainly add to the sparse literature currently available.

## Data Availability

The datasets generated during and analyzed during the current study are not publicly available due to confidentiality reasons but are available from the corresponding author on reasonable request.

## References

[1] Wilcox AJ, Weinberg CR, O’Connor JF (1988). Incidence of early loss of pregnancy. N Engl J Med.

[2] Trinder J, Brocklehurst P, Porter R, Read M, Vyas S, Smith L (2006). Management of miscarriage: expectant, medical, or surgical? Results of randomised controlled trial (miscarriage treatment (MIST) trial). BMJ.

[3] Graziosi GCM, Mol BWJ, Reuwer PJH, Drogtrop A, Bruinse HW, 4 (2004). Misoprostol versus curettage in women with early pregnancy failure after initial expectant management: a randomized trial. Hum Reprod.

[4] Creinin MD (2000). Randomized comparison of efficacy, acceptability and cost of medical versus surgical abortion. Contraception.

[5] Bagratee JS, Khullar V, Regan L, Moodley J, Kagoro H (2004). A randomized controlled trial comparing medical and expectant management of first trimester miscarriage. Hum Reprod.

[6] Kong GW, Lok IH, Yiu AK, Hui AS, Lai BP, Chung TK (2013). Clinical and psychological impact after surgical, medical or expectant management of first-trimester miscarriage – a randomised controlled trial. Aust N Z J Obstet Gynaecol.

[7] Bord I, Gdalevich M, Nahum R, Meltcer S, Anteby EY, Orvieto R (2014). Misoprostol treatment for early pregnancy failure does not impair future fertility. Gynecol Endocrinol.

[8] Mizrachi Y, Ben-Ezry E, Kleiner I (2020). Reproductive outcome after early pregnancy loss treated with misoprostol versus surgical aspiration. Reprod Biomed Online.

[9] Zhang J, Gilles JM, Barnhart K (2005). A comparison of medical management with misoprostol and surgical management for early pregnancy failure. N Engl J Med.

[10] Kim C, Barnard S, Neilson JP, Hickey M, Vazquez JC, Dou L (2017). Medical treatments for incomplete miscarriage. Cochrane Database Syst Rev.

[11] De Jonge ET, Makin JD, Manefeldt E, De Wet GH, Pattinson RC (1995). Randomised clinical trial of medical evacuation and surgical curettage for incomplete miscarriage. BMJ.

[12] Goldberg AB, Greenberg MB, Darney PD (2001). Misoprostol and pregnancy. N Engl J Med.

[13] Chung TK, Lee DT, Cheung LP, Haines CJ, Chang AM (1999). Spontaneous abortion: a randomized, controlled trial comparing surgical evacuation with conservative management using misoprostol. Fertil Steril.

[14] Reif P, Tappauf C, Panzitt T, Haas J, Lang U, Klaritsch P (2013). Efficacy of misoprostol in relation to uterine position in the treatment of early pregnancy failure. Int J Gynaecol Obstet.

[15] Kovavisarach E, Jamnansiri C (2005). Intravaginal misoprostol 600 mg and 800 mg for the treatment of early pregnancy failure. Int J Gynaecol Obstet.

[16] Tanha FD, Feizi M, Shariat M. Sublingual versus vaginal misoprostol for the management of missed abortion. J Obstet Gynaecol Res 36/3:525–32. 10.1111/j.1447-0756.2010.01229.x 20598032

[17] Saxena P, Salhan S, Sarda N (2004). Comparison between the sublingual and oral route of misoprostol for pre-abortion cervical priming in first trimester abortions. Hum Reprod.

[18] Odeh M, Tendler R, Kais M, Maximovsky O, Ophir E, Bornstein J (2010). Early pregnancy failure: factors affecting successful medical treatment. Isr Med Assoc J.

[19] Creinin MD, Huang X, Westhoff C, Barnhart K, Gilles JM, Zhang J (2006). Factors related to successful misoprostol treatment for early pregnancy failure. Obstet Gynecol.

[20] Weeks AD (2008). The retained placenta. Best Pract Res Clin Obstet Gynaecol.

[21] Greenbaum S, Wainstock T, Dukler D, Leron E, Erez O (2017). Underlying mechanisms of retained placenta: evidence from a population based cohort study. Eur J Obstet Gynecol Reproductive Biology.

[22] Männistö J, Mentula M, Bloigu A (2013). Medical versus surgical termination of pregnancy in primigravid women–is the next delivery differently at risk? A population-based register study. BJOG.

[23] Doubilet PM, Benson CB, Bourne T (2013). Society of radiologists in ultrasound multispecialty panel on early first trimester diagnosis of miscarriage and exclusion of a viable intrauterine pregnancy. Diagnostic criteria for nonviable pregnancy early in the first trimester. N Engl J Med.

[24] Chill HH, Malyanker N, Karavani G (2018). Association between uterine position and transvaginal misoprostol treatment for early pregnancy failure. J Obstet Gynaecol Res.

[25] Herman A (2000). Complicated third stage of labor: time to switch on the scanner. Ultrasound Obstet Gynecol.

[26] Herman A, Weinraub Z, Bukovsky I (1993). Dynamic ultrasonographic imaging of the third stage of labor: new perspectives into third stage mechanisms. Am J Obstet Gynecol.

[27] Harden MA, Walters MD, Valente PT (1990). Postabortal hemorrhage due to placenta increta: a case report. Obstet Gynecol.

[28] Wang YL, Weng SS, Huang WC (2019). First-trimester abortion complicated with placenta accreta: a systematic review. Taiwan J Obstet Gynecol.

[29] Belachew J, Cnattingius S, Mulic-Lutvica A, Eurenius K, Axelsson O, Wikstrom AK (2014). Risk of retained placenta in women previously delivered by caesarean section: a population-based cohort study. BJOG.

